# Implementation of genomic medicine for rare disease in a tertiary healthcare system: Mayo Clinic Program for Rare and Undiagnosed Diseases (PRaUD)

**DOI:** 10.1186/s12967-023-04183-7

**Published:** 2023-06-23

**Authors:** Filippo Pinto e Vairo, Jennifer L. Kemppainen, Carolyn R. Rohrer Vitek, Denise A. Whalen, Kayla J. Kolbert, Kaitlin J. Sikkink, Sarah A. Kroc, Teresa Kruisselbrink, Gabrielle F. Shupe, Alyssa K. Knudson, Elizabeth M. Burke, Elle C. Loftus, Lorelei A. Bandel, Carri A. Prochnow, Lindsay A. Mulvihill, Brittany Thomas, Dale M. Gable, Courtney B. Graddy, Giovanna G. Moreno Garzon, Idara U. Ekpoh, Eva M. Carmona Porquera, Fernando C. Fervenza, Marie C. Hogan, Mireille El Ters, Kenneth J. Warrington, John M. Davis, Matthew J. Koster, Amir B. Orandi, Matthew L. Basiaga, Adrian Vella, Seema Kumar, Ana L. Creo, Aida N. Lteif, Siobhan T. Pittock, Peter J. Tebben, Ejigayehu G. Abate, Avni Y. Joshi, Elizabeth H. Ristagno, Mrinal S. Patnaik, Lisa A. Schimmenti, Radhika Dhamija, Sonia M. Sabrowsky, Klaas J. Wierenga, Mira T. Keddis, Niloy Jewel J. Samadder, Richard J. Presutti, Steven I. Robinson, Michael C. Stephens, Lewis R. Roberts, William A. Faubion, Sherilyn W. Driscoll, Lily C. Wong-Kisiel, Duygu Selcen, Eoin P. Flanagan, Vijay K. Ramanan, Lauren M. Jackson, Michelle L. Mauermann, Victor E. Ortega, Sarah A. Anderson, Stacy L. Aoudia, Eric W. Klee, Tammy M. McAllister, Konstantinos N. Lazaridis

**Affiliations:** 1 Center for Individualized Medicine, Mayo Clinic, 200 First Street SW, Rochester, MN 55905 USA; 2 Department of Clinical Genomics, Mayo Clinic, Rochester, MN USA; 3 Office of Access Management, Mayo Clinic, Rochester, MN USA; 4 Illumina, Inc., San Diego, CA USA; 5 Center for Individualized Medicine, Mayo Clinic, Jacksonville, FL USA; 6 Center for Individualized Medicine, Mayo Clinic, Scottsdale, AZ USA; 7 Division of Pulmonary and Critical Care Medicine, Mayo Clinic, Rochester, MN USA; 8 Division of Nephrology and Hypertension, Mayo Clinic, Rochester, MN USA; 9 Division of Rheumatology, Mayo Clinic, Rochester, MN USA; 10 Department of Pediatric Rheumatology, Mayo Clinic, Rochester, MN USA; 11 Division of Endocrinology, Diabetes, Metabolism, and Nutrition, Department of Medicine, Mayo Clinic, Rochester, MN USA; 12 Division of Pediatric Endocrinology, Department of Pediatric and Adolescent Medicine, Mayo Clinic, Rochester, MN USA; 13 Division of Endocrinology, Mayo Clinic, Jacksonville, FL USA; 14 Division of Pediatric Allergy and Immunology, Mayo Clinic, Rochester, MN USA; 15 Division of Pediatric Infectious Diseases, Department of Pediatric and Adolescent Medicine, Mayo Clinic, Rochester, MN USA; 16 Division of Hematology, Department of Internal Medicine, Mayo Clinic, Rochester, MN USA; 17 Department of Clinical Genomics, Mayo Clinic, Phoenix, AZ USA; 18 Department of Clinical Genomics, Mayo Clinic, Jacksonville, FL USA; 19 Division of Nephrology, Mayo Clinic, Scottsdale, AZ USA; 20 Division of Gastroenterology and Hepatology, Mayo Clinic, Scottsdale, AZ USA; 21 Department of Family Medicine, Mayo Clinic, Jacksonville, FL USA; 22 Department of Medical Oncology, Mayo Clinic, Rochester, MN USA; 23 Department of Pediatric Gastroenterology, Mayo Clinic, Rochester, MN USA; 24 Division of Gastroenterology and Hepatology, Department of Medicine, Mayo Clinic, Rochester, MN USA; 25 Division of Pediatric Rehabilitation Medicine, Department of Physical Medicine and Rehabilitation, Mayo Clinic, Rochester, MN USA; 26 Department of Neurology, Mayo Clinic, Rochester, MN USA; 27 Division of Respiratory Medicine, Mayo Clinic, Scottsdale, AZ USA; 28 Department of Surgery, Mayo Clinic, Rochester, MN USA; 29 Department of Quantitative Health Sciences, Mayo Clinic, Rochester, MN USA

**Keywords:** Rare disease, Undiagnosed disease, Individualized medicine, Genomics, Genetic counseling

## Abstract

**Background:**

In the United States, rare disease (RD) is defined as a condition that affects fewer than 200,000 individuals. Collectively, RD affects an estimated 30 million Americans. A significant portion of RD has an underlying genetic cause; however, this may go undiagnosed. To better serve these patients, the Mayo Clinic Program for Rare and Undiagnosed Diseases (PRaUD) was created under the auspices of the Center for Individualized Medicine (CIM) aiming to integrate genomics into subspecialty practice including targeted genetic testing, research, and education.

**Methods:**

Patients were identified by subspecialty healthcare providers from 11 clinical divisions/departments. Targeted multi-gene panels or custom exome/genome-based panels were utilized. To support the goals of PRaUD, a new clinical service model, the Genetic Testing and Counseling (GTAC) unit, was established to improve access and increase efficiency for genetic test facilitation. The GTAC unit includes genetic counselors, genetic counseling assistants, genetic nurses, and a medical geneticist. Patients receive abbreviated point-of-care genetic counseling and testing through a partnership with subspecialty providers.

**Results:**

Implementation of PRaUD began in 2018 and GTAC unit launched in 2020 to support program expansion. Currently, 29 RD clinical indications are included in 11 specialty divisions/departments with over 142 referring providers. To date, 1152 patients have been evaluated with an overall solved or likely solved rate of 17.5% and as high as 66.7% depending on the phenotype. Noteworthy, 42.7% of the solved or likely solved patients underwent changes in medical management and outcome based on genetic test results.

**Conclusion:**

Implementation of PRaUD and GTAC have enabled subspecialty practices advance expertise in RD where genetic counselors have not historically been embedded in practice. Democratizing access to genetic testing and counseling can broaden the reach of patients with RD and increase the diagnostic yield of such indications leading to better medical management as well as expanding research opportunities.

**Supplementary Information:**

The online version contains supplementary material available at 10.1186/s12967-023-04183-7.

## Introduction

In the United States, rare disease (RD) is defined a condition, which affects fewer than 200,000 individuals, or about 1 in 1,600 people considering the current population. The European Union and the World Health Organization (WHO) define a disease as rare when it affects fewer than 1 in 2,000 individuals [1]. It is estimated that as many as 9,000 distinct RD exist and as much as 6% of the world population is affected by one of them [2]. About 80% of RD are suspected to be caused by genetic variations and, in contrast to what has been thought, a substantial proportion of patients may present with signs and/or symptoms during adulthood. With the increasing use of genomic testing such as exome- or genome-sequencing in the past decade, many individuals with undiagnosed conditions have been diagnosed with a RD [3].

In the past 5 years, RD has experienced steeply progress in scientific discovery, however limited and slow progress in therapeutics despite the passage of the 1983 *US Orphan Drug Act*. The act attempted to address the absence of financial incentives to develop therapies for RD by providing a system of tax credits, government grants, and assistance for relevant clinical research. Nevertheless, lack of awareness of RDs by doctors and health systems, absent or small registries and shortage of available biospecimens of patients with RD, limited funding, and scarce opportunities for blockbuster therapies have discouraged clinicians, researchers, policy makers, and pharmaceutical companies to significantly invest in RD [4]. In the recent years, widespread access to and use of social media, creation of patient support groups dedicated to RD, as well as technological innovation such as next generation sequencing have promoted better study and understanding of RD [5].

At Mayo Clinic, the Center for Individualized Medicine (CIM) created the Program for Rare and Undiagnosed Diseases (PRaUD)- a systematic, integrated, and enterprise-wide approach aimed to: (i) improve the triage of patients with RD to facilitate better clinical care in subspecialty practice including proper referrals to medical genetic specialists, as needed; (ii) transform the delivery of medical practice by establishing genomic-based clinical services for RD in close collaboration with subspecialty divisions/departments; (iii) promote research by developing registries and a biorepository for RD as well as further the scientific networking both intra- and extra- murally; and (iv) raise awareness of patients, families, healthcare professionals, and public about RD.

In the past decade, genetic testing has become more available among subspecialty clinical practices of tertiary medical centers beyond its traditional use within medical genetics departments. For example, genetic testing has been applied into the care of patients within nephrology [6], oncology [7], cardiology [8], and neurology [9] and gastroenterology/hepatology [10]. Given the impact of genetic testing, pre- and post-test genetic counseling as well as informed consent is recommended by national organizations and often required by insurance payers. Genetic counseling is an important part of the genetic testing process. It helps patients understand: (i) expectations and limitations of testing options; (ii) potential impact in clinical management; and (iii) consequences to family members. Nevertheless, because of significant shortage of genetic counselors, genetic testing and the counseling process must be optimized to reach more patients in need.

To support such scalability, we created the Genetic Testing and Counseling (GTAC) unit as part of PRaUD aiming to: (i) streamline and scale the genetic testing ordering process; (ii) improve access to genetic testing; (iii) enhance subspecialist provider engagement, education, and satisfaction; and (iv) increase genetic counselor productivity.

Herein, we share our experience and learned lessons with PRaUD as relate to RD patients seen in subspecialty practices but not evaluated by trained medical geneticists. We highlight clinical model implementation details, metrics, and outcomes as medical subspecialist care for patients with RD. Our experience with patients in diagnostic odyssey has already been reported and is not included in this publication [3].

## Methods

### Genomic clinics

PRaUD introduced the concept of *genomic clinics* for RD and implemented those in collaboration with 11 clinical divisions/departments. Prior to launching the genomic clinic, the leadership of PRaUD met and engaged with the leadership of each division/department (i.e., chair, practice chair, administrators) to discuss and design the process, outline expectations, coordinate operations and define a plan of complete integration as well as the responsibility of each division/department in applying genomic tests into routine practice. Each genomic clinic was led by a subspecialty physician champion in collaboration with the operations team of PRaUD. A physician-champion was appointed to serve as a super-user for each genomic clinic with the aims to better understand [1] the needs of these patients, and [2] the relevant gaps of practice. Moreover, the physician-champion was responsible to disseminate his/her experience of the genomic clinic to other practitioners of the relevant division/department.

The cohort of this study is comprised of patients with a suspected genetic component for their phenotype along with their available family members evaluated at one of Mayo Clinic campuses in Minnesota, Florida, and Arizona between December 2018 and December 2022. Patients were identified by the subspecialty healthcare providers from the partner divisions/departments. A list of the participating divisions/departments, respective phenotypes, and number of patients evaluated are depicted in Table 1. Demographic and clinical data were obtained by electronic health records (EHR) review. Age at onset of symptoms was determined as the age at which the first symptom or sign was noted by the patient or their family members.

**Table 1:** Divisions and clinical indications included in the Program for Rare and Undiagnosed Diseases (PRaUD)

Division	Clinical indication	Number of patients
Allergy and immunology	Primary Immune Deficiency (PID) and Auto-Inflammatory Syndromes	82
Endocrinology	Maturity Onset Diabetes of the Young (MODY), Early Onset Osteoporosis, Monogenic Obesity, Short Stature, Pituitary Adenoma	166
Gastroenterology and hepatology	Cholestatic Liver Disease, Cholangiocarcinoma, Hepatocellular Carcinoma, IBD (unresponsive to therapy)	54
Infectious diseases	Primary Immune Deficiency (PID)	4
Nephrology and hypertension	Glomerular, CAKUT, Tubulointerstitial Disease, Stones (rare forms), Cysts (rare forms), Electrolyte imbalance	425
Nephrology transplant	Glomerular, CAKUT, Tubulointerstitial Disease, Stones (rare forms), Cysts (rare forms), Electrolyte imbalance	86
Neurology	Neuromuscular Diseases, Leukodystrophy, Epilepsy, Alzheimer's and Frontotemporal Dementia, Peripheral Neuropathy	81
Oncology	Soft Tissue and Bone Sarcoma	64
Physical medicine and rehabilitation	Cerebral Palsy	3
Pulmonary and critical care medicine	ILD/TBD, Bronchiectasis	72
Rheumatology	Auto-inflammatory Syndromes, Polyarteritis Nodosa (PAN), VEXAS	115

*MODY* maturity onset diabetes of the young, *IBD* inflammatory bowel disease, *CAKUT* congenital anomalies of kidney and urinary tract, *ILD* interstitial lung disease, *TBD* telomere biogenesis disorders, *VEXAS* vacuoles, E1 enzyme, X-linked, autoinflammatory, somatic syndrome

### Genetic testing

Genomic DNA was isolated from whole blood or buccal swab samples. Targeted next generation sequencing (NGS) multi-gene panels and customized exome- or genome-based panels associated with specific phenotypes curated by the PRaUD team were performed at Clinical Laboratory Improvement Amendments (CLIA)-certified and College of American Pathologists (CAP)-accredited laboratories. For a subset of African/African American individuals with glomerulopathy, targeted analysis of the known *APOL1* (apolipoprotein L1) risk alleles was performed and for some individuals with tubulointerstitial disease, a targeted analysis for the common pathogenic variant in *MUC1* was done at the Broad Institute. Some individuals participating in research activities had exome sequencing done on a research basis at Mayo Clinic Medical Genome Facility in Rochester, MN, or genome sequencing done at an external commercial laboratory. Genomic data were subsequently analyzed by a clinical geneticist trained in genomic variant interpretation at Mayo Clinic. Reportable genetic variants found by research testing were confirmed in a CLIA-certified and CAP-accredited laboratory. Genetic variants were classified according to the 2015 American College of Medical Genetics and Genomics (ACMG)/Association for Molecular Pathology (AMP) and following updated recommendations [11].

### Genetic counseling

**Fig. 1:**
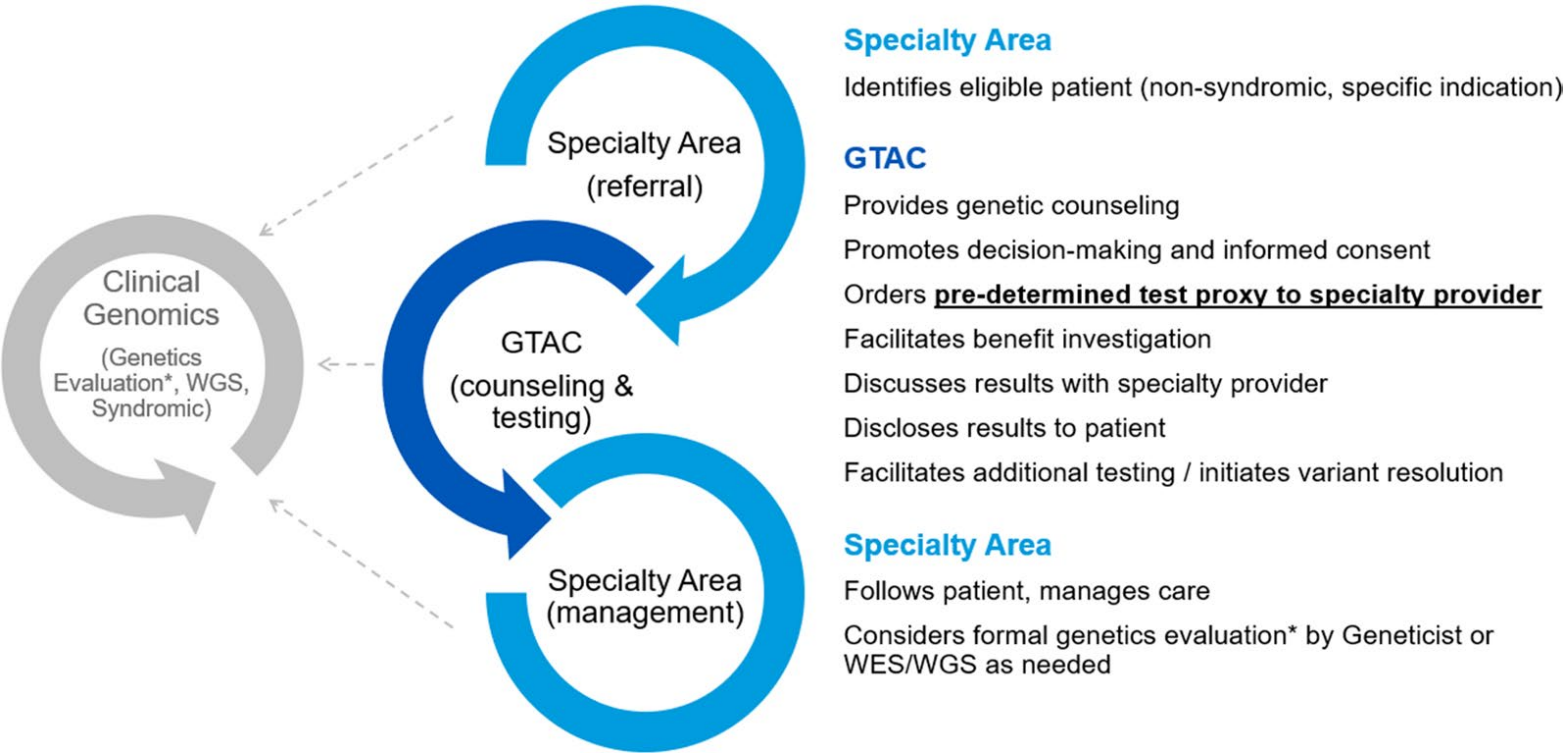
Genetic Testing And Counseling (GTAC) unit. The GTAC unit serves to facilitate genetic counseling for patients and genetic test ordering with specialty clinicians. It provides a streamlined approach to enhance access to focused genetic testing and counseling for identified conditions; reduced time for referrals; and just-in-time education for clinicians with limited genetic knowledge. Complex or syndromic cases requiring comprehensive genomic testing are referred to medical geneticists in the Department of Clinical Genomics

Genetic counseling was provided by the Genetic Testing And Counseling (GTAC) unit. The GTAC unit is comprised of genetic counselors, genetic counseling assistants (GCA), and genetic nurses, with medical geneticist physician oversight. The GTAC unit service is readily available at the time of patient’s need, usually offered within 48 h of patient’s referring subspecialty clinical appointment at Mayo Clinic (Fig. 1). The GTAC unit organizes the logistical aspects of the process, including appointment triage, medical and family history intake, and test requisition completion. The patient has a brief encounter (15–20 min) with a genetic counselor to understand the genetic testing process, ensure informed consent, and review personal and family implications of their results is provided. The GCAT unit tracks testing progress including insurance pre-authorization and sample receipt along with troubleshooting issues. When the genetic test report is received, the GTAC unit reviews the reported genetic variants and develops a plan of care with the referring subspecialty physician. A genetic counselor reviews results with the patient and/or family through a return-of-results video appointment and provides a written summary of results including additional recommendations, review of available guidelines and implications for the patient and family members.

### Operational support

The multidisciplinary planning and operational team of PRaUD incorporates a project manager, a program manager, and an operations manager to provide support and reduce barriers to implementation, including, but not limited to organizing meetings, engaging subspecialty physician champions and ancillary stakeholders, managing, and tracking timelines, developing workstreams, creating system orders and scheduling, training staff, creating databases/reporting, as well as assessing implementation outcomes. Clinical research coordinators consent and enroll patients to relevant research protocols and studies.

## Results

### Patient cohort

A total of 1152 patients without a confirmed genetic diagnosis prior to evaluation by the subspecialty physician champion were included in this study. The cohort was comprised of 50.3% female patients and 23.8% were under 18 years of age. Age at time of clinical genetic testing ranged from 1 to 87 years with a median age of 44 years. The largest group of clinical indications were from nephrology (six indications) followed by rheumatology, neurology, and endocrinology (five indications each) (Table 1). Positive family history for similar phenotypes were reported by 60.4% of the probands. A description of the referral age and age at onset of disease symptoms or signs per clinical indication can be found in Additional file 1: Tables S1 and Additional file 2: Table S2, respectively.

### Types of genetic testing

Targeted multi-gene panels were offered to 617 individuals (282 with kidney, 80 immunological, 59 endocrine, 59 neurological, 55 cancer, 42 GIH, 22 pulmonary, and 18 rheumatological phenotypes). Custom clinical exome-based panels were completed for 219 individuals (114 patients with kidney disease, 59 patients with recurrent fever/auto-inflammatory disease, 25 patients with interstitial lung disease/telomere biogenesis disorders, eight individuals with short stature, eight patients with early-onset osteopenia, three individuals with suspected MODY, and two patients with cholestasis). Custom clinical genome-based panels were done for a total of 89 individuals (50 in nephrology, 22 in rheumatology, 11 in endocrinology, 5 in pulmonary, and one in gastroenterology and hepatology). The type of genetic testing and genes to be included in the custom panels were decided by a multidisciplinary team of clinician and research experts on those phenotypes. Research consent was obtained from 407 individuals and research testing was performed for 117 individuals.

### Case solved status

Genetic testing was completed for 855 individuals. Overall, the solved rate was 14.1% (121/855) with a potential to increase to 17.5% (150/855) since some of the variants classified as of uncertain significance were deemed relevant by the multidisciplinary team and depending on variant phasing, segregation, or completion of focused clinical follow up tests such as biochemical and imaging could be reclassified as likely pathogenic. The solved status by divisions/departments and clinical phenotype is shown in Table 2 and a list of genetic variants per patient is in Additional file 3: Table S3.

**Table 2:** Solved statuses for different phenotypes

Division	Phenotype	Patients	Solved	Likely solved	Unsolved	Solved rate (minimum) %	Solved rate (potential) %
Allergy and immunology	PID	50	2	3	45	4.0	10.0
	Auto-inflammatory	14	1	0	13	7.1	7.1
Endocrinology	MODY	28	6	0	22	21.4	21.4
	Osteoporosis	24	2	1	21	8.3	12.5
	Monogenic obesity	7	0	0	7	0.0	0.0
	Short stature	30	6	1	23	20.0	23.3
	Pituitary adenoma	10	0	0	10	0.0	0.0
GIH	Cholestasis	5	0	1	4	0.0	20.0
	Cholangiocarcinoma	13	0	1	12	0.0	7.7
	IBD	15	0	0	15	0.0	0.0
	HCC	2	0	0	2	0.0	0.0
Infectious diseases	PID	2	0	0	2	0.0	0.0
	Auto-inflammatory	1	0	0	1	0.0	0.0
Nephrology	CAKUT	20	5	1	14	25.0	30.0
	Cysts	71	29	3	39	40.8	45.1
	Glomerular	187	39	9	139	20.9	25.7
	Electrolyte imbalance	14	0	1	13	0.0	7.1
	Stones	53	5	3	45	9.4	15.1
	Tubulointerstitial	9	4	0	5	44.4	44.4
Neurology	Epilepsy	17	4	0	13	23.5	23.5
	Neuromuscular	11	5	0	6	45.5	45.5
	Dementia	8	0	0	8	0.0	0.0
	Peripheral Neuropathy	4	0	0	4	0.0	0.0
Oncology	Sarcoma	42	0	0	42	0.0	0.0
Physical medicine	Cerebral palsy	2	0	0	2	0.0	0.0
Pulmonary	ILD/TBD	40	6	4	30	15.0	25.0
Rheumatology	Auto-inflammatory	77	3	1	73	3.9	5.2
	VEXAS	9	6	0	4	66.7	66.7
	IBD	1	0	0	1	0.0	0.0
	Osteoporosis	1	0	0	1	0.0	0.0

*GIH* gastroenterology and hepatology, *PID* primary immunodeficiency, *MODY* maturity onset diabetes of the young, *IBD* inflammatory bowel disease, *HCC* hepatocellular carcinoma, *ILD* interstitial lung disease, *TBD* telomere biogenesis disorders, *CAKUT* congenital anomalies of kidney and urinary tract, *VEXAS* vacuoles, E1 enzyme, X-linked, autoinflammatory, somatic syndrome

### Genetic testing and counseling (GTAC) unit workload and patient outcomes

**Fig. 2:**
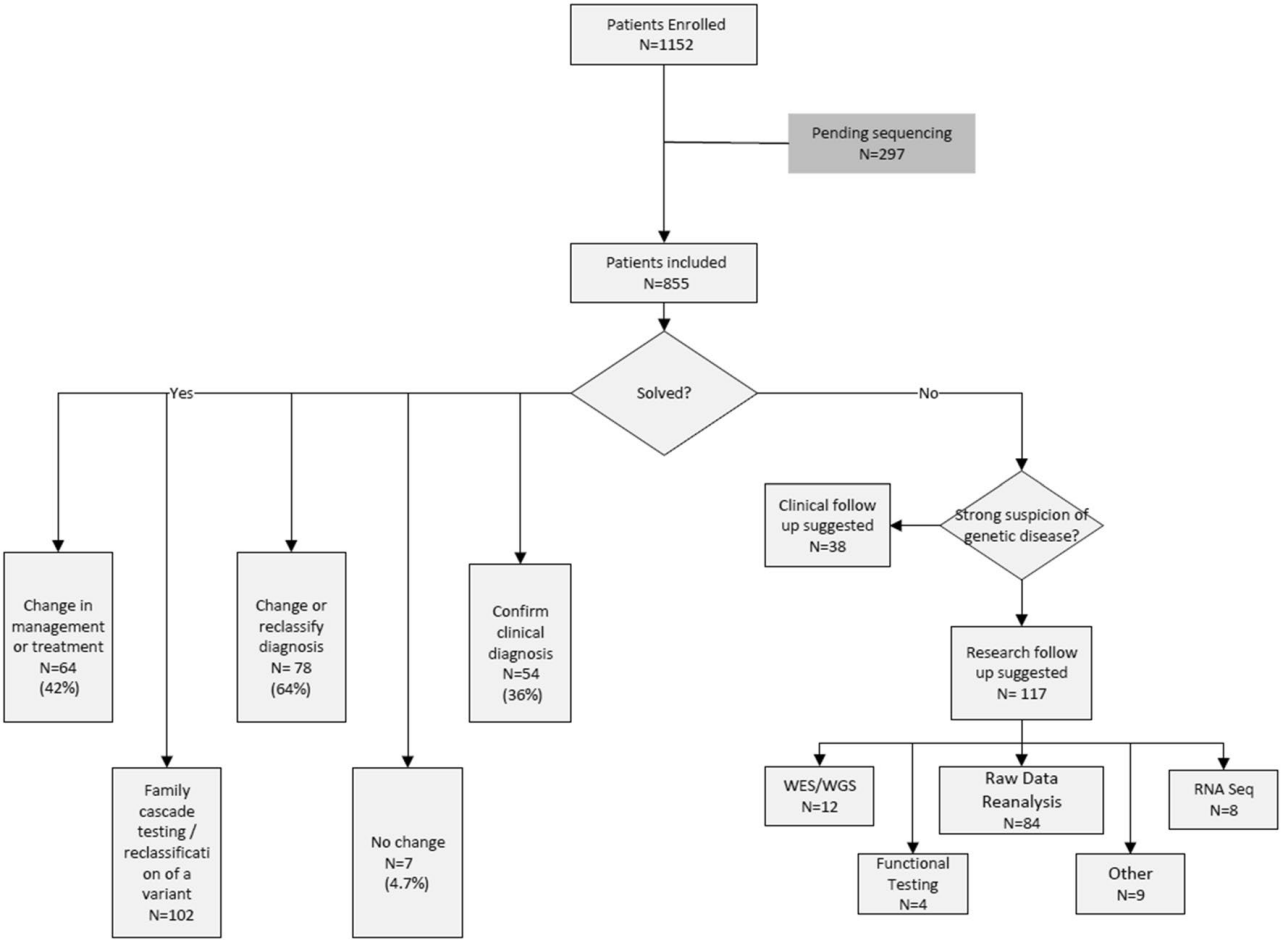
Summary of patient outcomes. Many patients with solved status (i.e., yes) led to several outcomes, which were grouped in categories such as (i) change in management or treatment; (ii) family cascade testing/reclassification of a variant; (iii) change or reclassification of diagnosis; (iv) confirmation of clinical diagnosis; and (v) no change. In some instances, more than one type of research test was completed for a patient, including: WES: exome sequencing, WGS: genome sequencing, targeted variant analysis, PCR, biochemical testing, or functional testing in other samples

GTAC provided pre-test telephone or video visits for 480 (41.7%) patients. The GTAC unit facilitated familial targeted variant testing for 102 family members, which aided in solving 25 (17%) cases due to variant segregation and additional clinical information from probands’ relatives. Genetic testing was essential in changing management for 42.7% (64/150) of the solved and the likely solved patients. A summary of patient outcomes is depicted in Fig. 2.

### Case examples

#### Case 1—Kidney transplant

A 38-year-old woman with proteinuria had a kidney biopsy showing focal segmental glomerulosclerosis (FSGS) lesion, which was thought to be suspected primary FSGS. She had persistent microscopic hematuria and variable levels of proteinuria. Her disease was refractory to multiple medications and progressed to end-stage kidney disease in her mid-30 s. GTAC facilitated a custom exome-based panel focused on kidney phenotypes, which detected a likely pathogenic variant in *COL4A3* (NM_000091.4:c. c.2126-1G > C), a gene associated with recessive and dominant forms of collagenopathy (also known as COL4A-related diseases or Alport disease), which was a good fit for patient’s kidney disease. Knowing that Alport disease might be associated with vision and hearing problems, she was referred to medical geneticist in the Department of Clinical Genomics for further specific evaluations. The genetic results confirmed that the patient’s disease was not primary FSGS, and thus, the recurrence risk post-transplant was low. Interestingly, a younger biological sister of the patient with no proteinuria nor hematuria was being evaluated as a potential donor. She had normal kidney function but was found to carry the same genetic variant as the patient. To this end, she was declined as a donor and counselled about risk of future kidney and extra-renal manifestations [12].

#### Case 2—Rheumatology

A 32-year-old man presented with history of recurrent ischemic cerebral infarcts and intermittent fevers. He had been clinically diagnosed with polyarteritis nodosa (PAN) in childhood when he presented with fever of unknown origin, neuropathy, aphthous ulcers, arthralgia, and hypertension. At age 29, he developed left-sided paresthesia, elevated inflammatory markers, and right pontine ischemic cerebral infarct. Thus, a diagnosis of central nervous system vasculitis was suspected. A custom, exome-based panel including genes associated with auto-inflammatory diseases detected a single heterozygous paternally inherited pathogenic variant in *ADA2* (NM_001282225.1: c.506G > A; p.(Arg169Gln)). The GTAC unit facilitated research enzymatic testing (at Hershield Laboratory, Duke University), which showed markedly decreased activity of ADA2 confirming the biochemical diagnosis of ADA2 deficiency (DADA2). Since a single variant was found, additional genetic testing was suggested. A high-density, exon-level, oligo array was performed, which detected a deletion encompassing the first exon of *ADA2.* Additional familial testing confirmed inheritance from the mother confirming this patient had two pathogenic variants in trans consistent with a molecular diagnosis. After the molecular and biochemical confirmation of DADA2, a TNF inhibitor was started with resolution of the fever and arthralgia and no other ischemic events have been reported [14].

#### Case 3—Immunology

A 13-year-old girl presented with weight loss and abdominal pain since early childhood. She had frequent visits to the emergency room monthly for abdominal pain and vomiting. She was evaluated for a number of gastrointestinal conditions including celiac disease and subsequent restrictive diet did not improve her symptoms. Due to suspected inflammatory bowel disease (IBD), a targeted multi-gene panel for primary immunodeficiencies, which includes the most common genetic causes for monogenic IBD was ordered and detected a likely pathogenic variant in *CTLA4* (NM_005214.4:c.401T > G; p.(Met134Arg))*.* This is a gene associated with immune dysregulation, auto-immunity, immunodeficiency, and lymphoproliferation, which was a good fit for patient’s signs and symptoms. GTAC facilitated familial cascade testing and confirmed that the variant was de novo in the proband. Based on the genetic testing results, abatacept—a fusion protein that includes part of *CTLA4* was prescribed. Soon after treatment, the patient reported improvement in weight, school attendance, pain, and the diet was liberalized.

#### Case 4—Endocrinology

A 16-year-old boy was newly diagnosed with diabetes and was using insulin 3–4 times a day, with no control of his blood sugar. His mother was diagnosed with diabetes at the age of 13. She had been on and off insulin in the past; however following pregnancy, insulin was resumed, and she has been on insulin ever since. His maternal uncle was diagnosed with diabetes at the age of 19 years and multiple other family members had a diabetes diagnosis and have been managed with insulin. A custom exome-based panel including genes associated with monogenic diabetes detected a likely pathogenic variant in *HNF1A* (NM_000545.5: c.526 + 1G > A) confirming the diagnosis of Maturity Onset Diabetes of the Young (MODY). Familial targeted testing was offered and confirmed the genetic diagnosis in the affected maternal relatives. Based on the genetic testing results, the proband was transitioned to the oral medication glipizide. The patient’s glucose levels improved, and insulin was discontinued.

## Discussion

In this paper, we report on PRaUD—an innovative program and clinical service model at Mayo Clinic aimed at integrating genomics-based care into subspecialty practices for patients with RD. Implementing genetic testing in practice, understanding, and applying such results in the era of next-generation sequencing is a complex task, necessitating specific skills and training for sequence variant interpretation as well communication and education of the healthcare provider, patient, and their family. Providing support for or against pathogenicity of a variant is an arduous and time-consuming process. Moreover, many subspecialty clinicians and researchers lack the time, expertise, appropriate tools or experience to interpret a genetic variant correctly.

The process of ordering genetic testing and interpreting the results is complex and burdensome with barriers that often limit the use of such by a subspecialist with no formal training in medical genetics. Furthermore, insurance companies and other payers usually request providers to demonstrate clinical utility of this type of testing in subspecialty clinics other than in a medical genetics department setting. To overcome these challenges, PRaUD developed an integrated team and process to support subspecialty champion physicians to the use genetic testing in patient care. Moreover, the PRaUD team of clinicians, genetic counselors, along with research scientists was integral in evaluating and validating genetic variants derived from multi-gene panel testing leading to increased access to testing, better diagnosis, improved patient care, new knowledge, and academic output. Based on this multidisciplinary approach including the ordering subspecialty providers, clinical and research follow-up studies are suggested and facilitated to validate genetic findings in the context of the patient’s phenotype. If targeted genetic testing results in a negative or inconclusive result, then patients with compelling phenotypes were referred to the Department of Clinical Genomics for further evaluation by a board-certified medical geneticist and offered comprehensive genomic tests such as exome or genome sequencing.

The GTAC unit provides resources to assist clinicians with genetic test identification, review, and optimization; genetic counseling access; end-to-end process development; genetic test education and interpretation; testing and patient tracking; research protocol facilitation and collaboration; registry and biorepository services; and participation in clinical trials. Additionally, GTAC is the contact point for RD patient advocacy groups and foundations.

The case vignettes presented above highlight the importance of integrating genomic testing into the standard clinical care of subspecialty clinics. The genetic findings not only impacted the probands seeking care but also other family members. Case 1 proves the potential of genetic testing for unaffected individuals who otherwise would be cleared for solid organ donation without knowing the increased risk of developing the inherited familial disease in the future as well as preventing a wasteful organ transplantation for both the donor and recipient. Case 2 highlights the need of a multidisciplinary team in the care of individuals going through the genetic testing process. It is imperative to understand the limitations of the different types of genetic testing. For this case, a single exon deletion was missed by the technology used for the custom exome-based panel. With that knowledge, the GTAC unit suggested a more in-depth analysis for deletions/duplications using a targeted microarray, which ultimately detected the previously missed allele. Moreover, the integration of a research component into the clinical practice was able to provide a biochemical diagnosis for that individual allowing for the prescription of a disease-specific medication. Clinical multi-gene panels or exome sequencing for IBD non-responding to standard medical therapy has an overall low diagnostic rate at 5–10% [3] with a higher solved rate in individuals with very-early onset of the disease. In Case 3, the genetic results were impactful in determining a targeted medication, which speedily resolved the patient’s symptoms and improved her quality of life. Case 4 emphasizes the importance of genetic testing for common phenotypes. It is well known that diabetes has a strong genetic component, but monogenic causes are rare, ranging from 1 to 5% of pediatric and young adult populations [14]. With multiple affected individuals at early ages in that family, MODY was suspected. Importantly, with the confirmation of MODY, affected individuals could be transitioned from daily, multiple insulin shots to an oral medication, which improved the glucose levels and positively impacted quality of life and clinical outcomes.

Over the course of a four-year period, PRaUD integrated genetic testing into subspecialty clinical practices for 29 clinical indications across 11 divisions/departments. The range of time for implementation was influenced by champion subspecialty physician availability, development of division/department protocol, leadership support, and IT technical challenges (*Rohrer Vitek, personal communication*).

Advances in telemedicine allows for virtual units to operate in centralized sites and offer the service at remote locations. Almost half of pre-test and over 95% of posttest GTAC unit appointments utilized virtual appointments. This model can allow flexibility to have staff of this unit working remotely, which increases genetic counselors’ recruitment and retention in a competitive market. The GTAC unit model continues to be refined to scale and accommodate continued growth in genetic testing, ensure a standardized process, and maintain direct collaboration with the ordering subspecialty physicians to appropriately incorporate the genetic testing results within the care plan of the patients and their family. Given that genomic testing is accurate, scalable, and now affordable, we recognized a great opportunity to incorporate this diagnostic tool into the subspecialty clinical practice at large. It was an imperative for us to apply genomic tests broadly to benefit RD patients because of the evidence of such testing to diagnose and improve management of these diseases.

Importantly, the development of PRaUD promoted diffusion and expansion of genetic testing services for RD throughout our tertiary healthcare system. Noteworthy, 9.5% of patients (81/855)  evaluated at the genomic clinics of PRaUD were subsequently referred to the Department of Clinical Genomics for care either directly after triage of the patient’s history and clinical data or following multi-gene panel results were unrevealing.

## Conclusions

We describe a systematic approach to enable clinical divisions/departments at a tertiary healthcare system to utilize genomic medicine in diagnostic and therapeutic selection for RD in subspecialty practices. As PRaUD expands it bridges a critical genetic testing and counseling access gap. Further, it enables the democratization of genetic expertise to subspecialty physicians and maintaining continuity of care, while also promoting referrals for those patients who would most benefit from further evaluation with trained medical geneticists in the Department of Clinical Genomics. Moreover, improved access to genetic counseling and testing via a hybrid telehealth service can increase diagnostic yield, reduce time to diagnosis, and expand reach for RD indications where testing has been underutilized; ultimately positively impacting a patient’s care. Finally, providing additional biorepository collections for research enhances the opportunities for future discovery in RD.

## Supplementary Information

**Additional file 1: Table S1.** Overview of clinical indications of PRaUD genomic clinics and referral age of patients.

**Additional file 2: Table S2.** Overview of clinical indications of PRaUD genomic clinics and age at onset of disease symptom or signs.

**Additional file 3: Table S3.** Summary of genetic variants of PRaUD genomic clinics per patient.

## Data Availability

The datasets generated and/or analyzed during the current study are not publicly available but are available from the corresponding author on reasonable request.

## Abbreviations

ACMG: American College of Medical Genetics and Genomics
AMP: Association for molecular pathology
CAP: College of American pathologists
CIM: Center for individualized medicine
CLIA: Clinical laboratory improvement amendments
DADA2: ADA2 deficiency
EDS: Ehlers-Danlos syndrome
FSGS: Focal segmental glomerulosclerosis
GTAC: Genetic testing and counseling unit
HCC: Hepatocellular carcinoma
IBD: Inflammatory bowel disease
ILD: Interstitial lung disease
MODY: Maturity onset diabetes of the young
PAN: Polyarteritis nodosa
PID: Primary immunodeficiency
PRaUD: Program for rare and undiagnosed diseases
RD: Rare disease
RUD: Rare and undiagnosed diseases
TBD: Telomere biogenesis disorders
VEXAS: Vacuoles, E1 enzyme, X-linked, autoinflammatory, somatic syndrome
