# Correction: Implementation of genomic medicine for rare disease in a tertiary healthcare system: Mayo Clinic Program for Rare and Undiagnosed Diseases (PRaUD)

**DOI:** 10.1186/s12967-024-05185-9

**Published:** 2024-04-30

**Authors:** Filippo Pinto e Vairo, Jennifer L. Kemppainen, Carolyn R. Rohrer Vitek, Denise A. Whalen, Kayla J. Kolbert, Kaitlin J. Sikkink, Sarah A. Kroc, Teresa Kruisselbrink, Gabrielle F. Shupe, Alyssa K. Knudson, Elizabeth M. Burke, Elle C. Loftus, Lorelei A. Bandel, Carri A. Prochnow, Lindsay A. Mulvihill, Brittany Thomas, Dale M. Gable, Courtney B. Graddy, Giovanna G. Moreno Garzon, Idara U. Ekpoh, Eva M. Carmona Porquera, Fernando C. Fervenza, Marie C. Hogan, Mireille El Ters, Kenneth J. Warrington, John M. Davis, Matthew J. Koster, Amir B. Orandi, Matthew L. Basiaga, Adrian Vella, Seema Kumar, Ana L. Creo, Aida N. Lteif, Siobhan T. Pittock, Peter J. Tebben, Ejigayehu G. Abate, Avni Y. Joshi, Elizabeth H. Ristagno, Mrinal S. Patnaik, Lisa A. Schimmenti, Radhika Dhamija, Sonia M. Sabrowsky, Klaas J. Wierenga, Mira T. Keddis, Niloy Jewel J. Samadder, Richard J. Presutti, Steven I. Robinson, Michael C. Stephens, Lewis R. Roberts, William A. Faubion, Sherilyn W. Driscoll, Lily C. Wong-Kisiel, Duygu Selcen, Eoin P. Flanagan, Vijay K. Ramanan, Lauren M. Jackson, Michelle L. Mauermann, Victor E. Ortega, Sarah A. Anderson, Stacy L. Aoudia, Eric W. Klee, Tammy M. McAllister, Konstantinos N. Lazaridis

**Affiliations:** 1 Center for Individualized Medicine, Mayo Clinic, 200 First Street SW, Rochester, MN 55905 USA; 2 Department of Clinical Genomics, Mayo Clinic, Rochester, MN USA; 3 Office of Access Management, Mayo Clinic, Rochester, MN USA; 4 Illumina, Inc., San Diego, CA USA; 5 Center for Individualized Medicine, Mayo Clinic, Jacksonville, FL USA; 6 Center for Individualized Medicine, Mayo Clinic, Scottsdale, AZ USA; 7 Division of Pulmonary and Critical Care Medicine, Mayo Clinic, Rochester, MN USA; 8 Division of Nephrology and Hypertension, Mayo Clinic, Rochester, MN USA; 9 Division of Rheumatology, Mayo Clinic, Rochester, MN USA; 10 Department of Pediatric Rheumatology, Mayo Clinic, Rochester, MN USA; 11 Division of Endocrinology, Diabetes, Metabolism, and Nutrition, Department of Medicine, Mayo Clinic, Rochester, MN USA; 12 Division of Pediatric Endocrinology, Department of Pediatric and Adolescent Medicine, Mayo Clinic, Rochester, MN USA; 13 Division of Endocrinology, Mayo Clinic, Jacksonville, FL USA; 14 Division of Pediatric Allergy and Immunology, Mayo Clinic, Rochester, MN USA; 15 Division of Pediatric Infectious Diseases, Department of Pediatric and Adolescent Medicine, Mayo Clinic, Rochester, MN USA; 16 Division of Hematology, Department of Internal Medicine, Mayo Clinic, Rochester, MN USA; 17 Department of Clinical Genomics, Mayo Clinic, Phoenix, AZ USA; 18 Department of Clinical Genomics, Mayo Clinic, Jacksonville, FL USA; 19 Division of Nephrology, Mayo Clinic, Scottsdale, AZ USA; 20 Division of Gastroenterology and Hepatology, Mayo Clinic, Scottsdale, AZ USA; 21 Department of Family Medicine, Mayo Clinic, Jacksonville, FL USA; 22 Department of Medical Oncology, Mayo Clinic, Rochester, MN USA; 23 Department of Pediatric Gastroenterology, Mayo Clinic, Rochester, MN USA; 24 Division of Gastroenterology and Hepatology, Department of Medicine, Mayo Clinic, Rochester, MN USA; 25 Division of Pediatric Rehabilitation Medicine, Department of Physical Medicine and Rehabilitation, Mayo Clinic, Rochester, MN USA; 26 Department of Neurology, Mayo Clinic, Rochester, MN USA; 27 Division of Respiratory Medicine, Mayo Clinic, Scottsdale, AZ USA; 28 Department of Surgery, Mayo Clinic, Rochester, MN USA; 29 Department of Quantitative Health Sciences, Mayo Clinic, Rochester, MN USA

**Correction: Journal of Translational Medicine (2023) 21:410** 10.1186/s12967-023-04183-7

Following publication of the original article [1], we have been notified by the authors that Additional files were not published correctly (Tables S1–S3). Also, declarations were not published correctly.

They are now as follows:

**Ethics approval and consent to participate**

The Mayo Clinic Institutional Review Board granted a waiver of consent for all the available clinical data of the electronic health records of the patients included in the study. All individuals participating in research activities provided written informed consent to a study approved by the Mayo Clinic Institutional Review Board (IRB#: 19-003389).

**Consent for publication**

Not applicable.

They should be as follows:

**Ethics approval and consent to participate**

Ethics approval for this retrospective study was granted by the Mayo Clinic Institutional Review Board (IRB#: 19-003389). The IRB provided review and approval for the use of all the available clinical data of the electronic health records of the patients included in this study. All individuals participating in prior research activities mentioned in this study provided written informed consent to a study approved by the Mayo Clinic Institutional Review Board.

**Consent for publication**

Consent for publication was obtained from the individuals highlighted in the Case Examples section.

The original article was updated.
